# Proof of principle for bevacizumab activity in desmoid-type fibromatosis

**DOI:** 10.1186/s13569-016-0045-3

**Published:** 2016-04-04

**Authors:** Viktor Grünwald, Florian Länger, H. J. Raatschen, Andreas Beilken

**Affiliations:** Clinic for Hematology, Hemostasis, Oncology and Stemcelltransplantation, Medical School Hannover, Carl-Neuberg-Str. 1, Hannover, Germany; Department of Pathology, Medical School Hannover, Hannover, Germany; Department of Radiology, Medical School Hannover, Hannover, Germany; Department of Pediatric Hematology and Oncology, Medical School Hannover, Hannover, Germany

**Keywords:** Aggressive fibromatosis, Desmoid, Bevacizumab, Anti-angiogenic therapy

## Abstract

**Background:**

Desmoid-type fibromatosis (DF) is a rare disease, which often occurs in young adults. Medical treatment is an important option in the treatment algorithm of DF. Different chemotherapeutic regimens showed clinical activity in DF, but overall treatment tolerability remains poor for this patient cohort. Novel approaches investigated tyrosine kinase inhibitors in DF, but tolerability remained an issue.

**Case presentation:**

We treated a patient with progressive DF after failure of chemotherapy for 1 year with singe agent bevacizumab. He achieved a symptomatic and radiologic response while attainning excellent tolerability.

**Conclusions:**

This is the first report on single agent bevacizumab in DF, which showed both, good tolerability and efficacy in our patient, thereby warranting future trials in DF.

## Background

Desmoid-type fibromatosis (DF) is a rare disease with an annual incidence of 3–4 cases per 1 million inhabitants, which is characterized by a variable and unpredictable clinical course. DF may occur at varying ages, but the majority of patients are diagnosed as young adults with a peak incidence at approximately 30 years [1]. Only a minority of 5–10 % of DF cases is associated with the familial adenomatous polyposis (FAP), an inherent disease with APC germ line mutation [2, 3].

Per definition, DF is a benign but clonal fibroblastic proliferation. It lacks the ability to form metastases, but its high local recurrence rate debilitates patients and affects their quality of life [4]. Therefore the choice of therapy should be made after interdisciplinary counseling, tailoring the treatment to the patients need. Because the natural course of the disease varies, different groups recommend active surveillance as the primary approach to these patients [3, 5]. Upon progression, treatment may consist of medical treatment, surgery or radiotherapy.

Discussing the merits of therapy also includes its major limitation, which in the case of surgery is the loss of function or its mutilating character. While medical treatment is associated with chronic exposure and toxicity may limit its prolonged use, radiotherapy offers some advantage with a single exposure, but the risk of secondary neoplasias is of specific interest in this non-malignant disease of mainly young adults [3].

Medical treatment is an option in progressive DF, which is used in patients who would require mutilating surgery or in the recurrent setting [4]. The agents used may vary from anti-inflammatory agents to chemotherapeutics. Methotrexate (MTX)/vinblastine (VBL) is a standard treatment approach in DF, which is intended to be given for a treatment duration of 1 year [3]. However, despite the good tolerability in metastatic cancers, MTX/VBL causes significant toxicity in DF, which hampers the continuation of treatment and requires treatment adjustments frequently [3]. In a recent pediatric phase II study only 50 % of patients finished the pre-specified treatment duration, and 64 % grade 3/4 toxicity was reported [6].

As an alternative approach, a recent study reported on the efficacy of sorafenib in DF. An objective response rate (ORR) of 25 % was achieved, indicating explicit efficacy in DF [7]. The median duration of sorafenib treatment was 5 months. The median daily dose of sorafenib was only 200 mg, which is ¼ of the standard dose in metastatic cancers (800 mg/day), indicating that distinct patient populations may exert distinct tolerability of treatment. Clearly, toxicity is a major theme for the choice of treatment in DF.

## Case presentation

### Description of the case

**Fig. 1:**
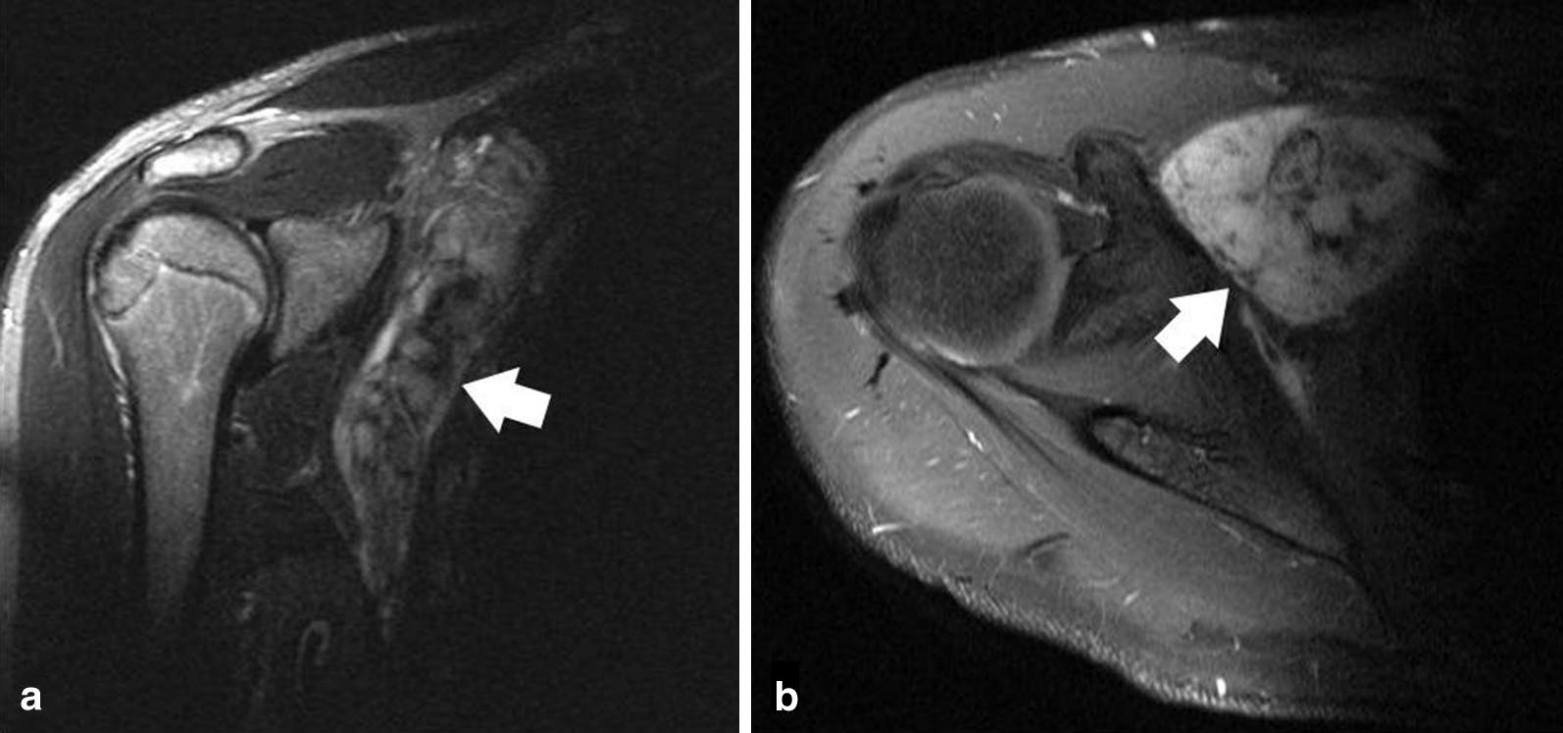
MRI of the right shoulder showing the tumor extent at the time of initial diagnosis in proton density (PD)-weighted (**a**) and T1-weighted contrast-enhanced (**b**) sequences. An inhomogeneously enhancing, partially fibrotic tumor (*white arrows*) originating from the right thoracic wall is clearly visible consistent with a desmoid-type fibromatosis

We report on a case of a 16-year old male patient who was diagnosed with DF of the right upper thorax in 2011 (Fig. 1). The primary approach consisted of partial tumor resection for functional preservation. Systemic therapy with MTX/VBL was started after completion of surgery in order to maintain tumor control. However, MTX-associated nausea and fatigue led to discontinuation of MTX after 6 months of treatment. Instead, sulfonylurea had been added to VBL, but again, nausea limited the applicability of this combination and treatment was provided as single agent sulfonylurea.

Two years after the initial resection, a symptomatic tumor recurrence was detected while receiving medical treatment. Local recurrence involved the right brachial plexus, which was incompletely resected for the purpose of function preservation.

Systemic therapy was reconvened with MTX and vinorelbine, which achieved symptomatic improvement of pain with disease stabilization as best response on MRI. However, the treatment was not sustainable because of grade 1 nausea and fatigue and led to treatment discontinuation after 4 months.

Within 4 months, the patient complained of recurrence of grade 1 tumor pain and the MRI showed gradual tumor growth. At this time, radiotherapy was not considered appropriate given the young age and the proximity to the plexus nerves, instead medical treatment was discussed with the patient. Re-challenge with chemotherapy was not preferred by the patient, and an individual approach to medical treatment was sought. Based on the hyperperfusion of the patient’s tumor and the promising results of sorafenib in DF, we implied future treatment with bevacizumab 15 mg/kg q3wks., given the excellent tolerability profile of this agent.

**Fig. 2:**
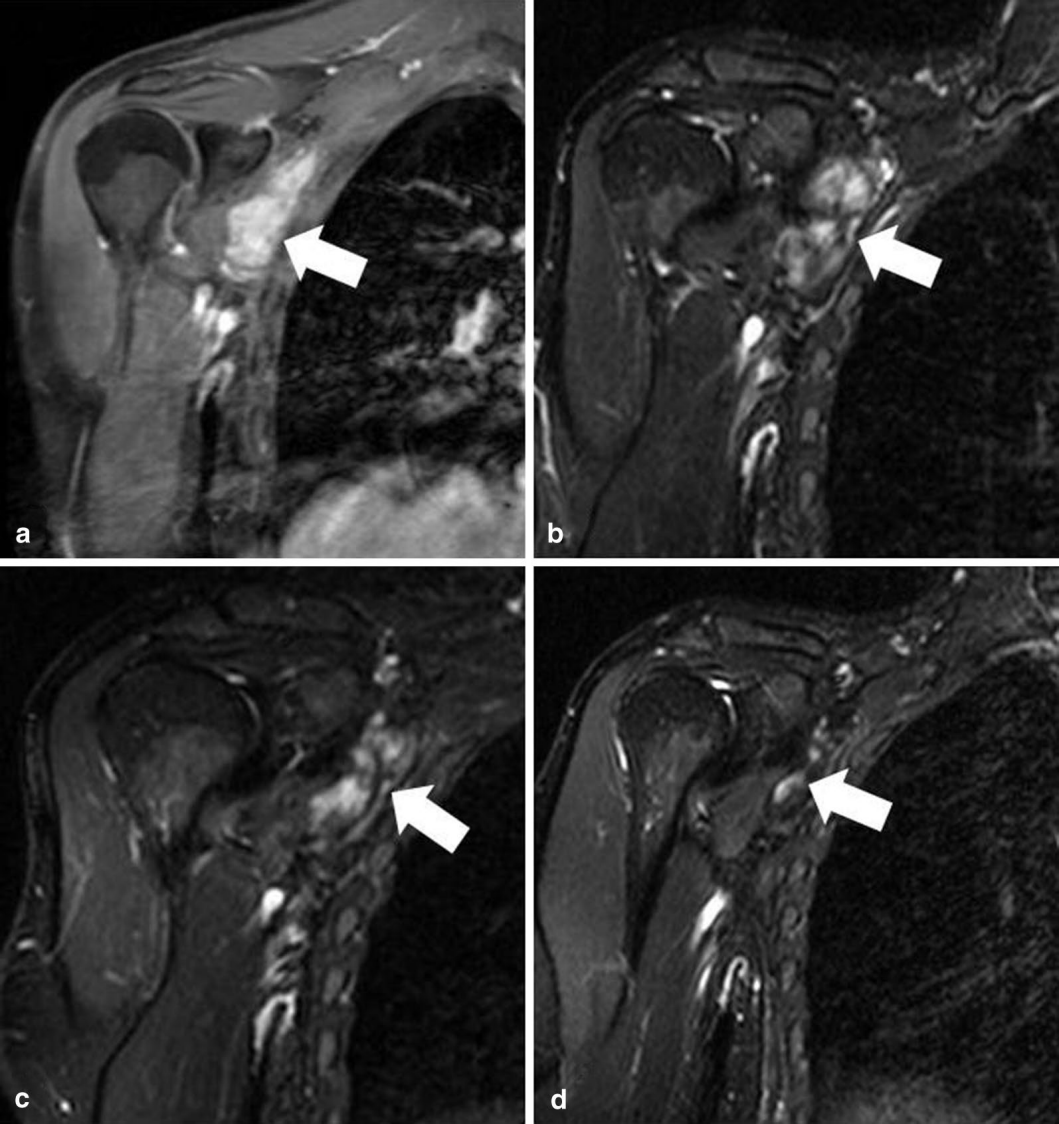
T2-weighted MRI of the right shoulder after second tumor resection and prior to bevacizumab treatment (**a**), as well as 6 weeks (**b**), 4 months (**c**) and 14 months (**d**) after treatment initiation, showing a continuous tumor shrinkage over time (*white arrows*)

During the course of treatment, tumor pain disappeared and the patient experienced functional improvement. Initial radiological response was detected after 1 month, with a tumorshrinkage of 16 %. Treatment was continued for a total duration of 12 months with a further decrease in tumor diameter of 38 % (Fig. 2). Further follow-up is performed by 3 months intervals by MRI and clinical visits. The first post-therapy scan confirmed tumor response in our patient, indicating sustained clinical activity.

## Discussion

Desmoid-type fibromatosis is a rare disease, which often occurs in young adults. Its lack of metastatic potential requires a distinct approach to treatment. Patients are not limited in their survival expectation, which renders long-term morbidity as a key factor for the process of shared decision-making. Medical treatment is an option for patients facing mutilating surgery. MTX/VBL with weekly applications is considered a well-tolerated agent in cancer patients. However, the treatment may be differentially anticipated in patients with DF, which is reflected by the high rate of treatment discontinuation in current patient series [3].

VEGFR inhibitors, such as sorafenib, exert an interesting activity in DF. However, these agents are associated with a high rate of grade 3/4 adverse events in >70 % of patients [8], and therefore represent an alternative for symptomatic patients who experience a DF-associated decrease in their daily activities.

Medical treatment is often chronic and requires long-term exposure, which renders VEGRF inhibitors not an ideal match for most patients. Based on the mechanism of action and the proof of principle by this class of agents, bevacizumab seems to be an attractive alternative with only a fraction of grade 3/4 adverse events (44 %) [9]. However, safety of bevacizumab is not well studied in children and adolescent patients and therefore should be used with caution in this population.

Our case report supports this notion with tumor shrinkage of DF and symptomatic improvement. The excellent tolerability of bevacizumab fits the needs of DF patients in many ways. The young age of most patients puts them into a scenario, where function and well-being is essential in their daily life. Our patient graduated successfully from secondary school while receiving bevacizumab treatment, indicating the excellent tolerability of this treatment.

Further studies with bevacizumab are warranted and should be pursuit in DF patients.

## Conclusion

Medical treatment of DF remains divers. While the majority of patients receive chemotherapy for tumor control, hormone ablation is used in selected cases, such as abdominal wall DF in young women. Novel approaches employ tyrosine kinase inhibitors, but given the vulnerability of this patient population, novel approaches are needed. In our patient bevacizumab achieved excellent tumor control with only a fraction of adverse events compared to conventional chemotherapy. Given the balance between efficacy and tolerability, bevacizumab may represent a suitable well-tolerated agent in DF and should be further explored in clinical trials.

## Consent

Informed consent was obtained from the patient for publication of this case report and images.
